# Association between interleukin 6 -174 G/C promoter gene polymorphism and runners' responses to the dietary ingestion of antioxidant supplementation based on pequi (*Caryocar brasiliense* Camb.) oil: a before-after study

**DOI:** 10.1590/1678-4685-GMB-2015-0299

**Published:** 2016-10-10

**Authors:** Ana Luisa Miranda-Vilela, Ieler Ferreira Ribeiro, Cesar Koppe Grisolia

**Affiliations:** 1 Department of Genetics and Morphology, Institute of Biological Sciences, Universidade de Brasilia, Brasilia, DF, Brazil.; 2 Faculty of Medicine, Faculdades Integradas da União Educacional do Planalto Central (Faciplac), Campus Gama, Brasília, DF, Brazil.; 3 Unieuro Centro Universitário, Brasilia, DF, Brazil.

**Keywords:** antioxidant supplementation, exercise-induced oxidative damage, inflammatory markers, nutrigenetics, nutrigenomics

## Abstract

Exercise is a double-edged sword: when practiced in moderation, it increases the expression of antioxidant enzymes, but when practiced strenuously it causes oxidative stress and cell damage. In this context, polymorphisms in the interleukin (IL)-6 gene should be investigated better because they can influence performance, at least in exercise that generates oxidative stress and leads to muscular injuries with consequent inflammation. In this work, we investigated the influence of IL-6 –174 G/C polymorphism on tissue damage and inflammation markers, lipid peroxidation, hemogram and lipid profile of runners before and after ingestion of 400 mg of pequi oil in capsules supplied daily for 14 consecutive days. The IL-6 genotypes were associated with significant differences in lipid peroxidation, with the CC mutant having lower values. There were also significant differences among these genotypes in the response to supplementation with pequi oil, exercise-induced damage and C-reactive protein (CRP) levels. The best protection against damage was observed with the heterozygous genotype. Although the CC genotype showed an increase in CRP levels after supplementation, the lack of a positive correlation between triglycerides and high-sensitivity CRP in this mutant genotype after supplementation indicated a protective effect of pequi. These findings deserve further investigation, particularly with regard to the quantification of circulating IL-6 concentrations.

## Introduction

Regular physical activity, apart from enhancing the expression of antioxidant enzymes, also induces a systemic increase in many cytokines with anti-inflammatory properties that protect against chronic disorders associated with low-grade systemic inflammation (Gomez-Cabrera *et al*., 2008; Colombini *et al*., 2011; Miranda-Vilela, 2012). Similarly, micro-injuries to skeletal muscle, resulting from regular exercise, lead to the recruitment of cytokines such as interleukin-1 beta (IL-1β) and tumor necrosis factor-alpha (TNF-α) that initiate and regulate the repair process. The long-term anti-inflammatory effect of exercise is also mediated by muscle-derived interleukin 6 (IL-6) (Colombini *et al*., 2011), which is involved in immune function, muscle repair and hypertrophy following exercise-induced damage (Eynon *et al*., 2011a). IL-6 stimulates circulating anti-inflammatory IL-1Ra and IL-10 and inhibits the production of pro-inflammatory TNF-α (Colombini *et al*., 2011).

In contrast to regular exercise, strenuous exercise or training above habitual intensity can lead to oxidative stress through the enhanced formation of reactive oxygen and nitrogen species (RONS), causing muscle injuries and inflammation that can compromise performance and potentially increase the future risk of cardiovascular disease (CVD) in athletes (Miranda-Vilela, 2012; Miranda-Vilela *et al*., 2011a, 2012). Strenuous exercise not only induces lipid peroxidation, but also promotes inflammation, changes in the immune cell count and the release of acute phase proteins such as C-reactive protein (CRP) (Oleto *et al*., 2011; Miranda-Vilela *et al*., 2009a, 2012; Miranda-Vilela, 2012). The synthesis of CRP is, in turn, regulated by cytokines, mostly IL-6 (Moleres *et al*., 2009); chronically elevated levels of IL-6 are associated with vascular smooth muscle growth, increased production of acute phase protein and effects on lipid and lipoprotein metabolism, all of which can contribute to an increased risk of CVD (Shen *et al*., 2008; Gan *et al*., 2013). Furthermore, an increase in the circulating levels of cytosolic proteins such as aspartate aminotransferase (AST), alanine aminotransferase (ALT) and creatine kinase (CK) after exercise reflects cellular injury and can be used as markers for exercise-induced damage (Akimoto *et al*., 2010; Miranda-Vilela 2012; Miranda-Vilela *et al*., 2012).

Physical exercise is thus a double-edged sword: when regularly practiced in moderation, it increases the expression of antioxidant enzymes and should be considered an antioxidant, but when practiced strenuously it causes oxidative stress and cell damage (Gomez-Cabrera *et al*., 2008), possibly leading to overtraining syndrome (Miranda-Vilela *et al*., 2011a; Miranda-Vilela, 2012). These observations have led to research into whether antioxidant supplementation could prevent the damaging effects of enhanced production of RONS in response to exercise, thereby improving athletic performance (Miranda-Vilela *et al*., 2011a; Miranda-Vilela, 2012). In this context, pequi oil, a carotenoid-rich oil extracted from the pulp of pequi (*Caryocar brasiliense* Camb.), a typical fruit found in the Brazilian Cerrado, has been shown to have anti-inflammatory properties, besides reducing arterial pressure, exercise-induced anisocytosis and DNA and tissue damage (Miranda-Vilela *et al*., 2009a,b, 2010, 2011a,b).

In addition to its natural antioxidants, pequi oil is composed mainly of oleic (51.37-55.87%) and palmitic (35.17-46.79%) fatty acids that modulate the triglyceride (TG):cholesterol ratio in postprandial triglyceride-rich lipoprotein (TRL) (Miranda-Vilela *et al*., 2009a,c). Postprandial and intestinally produced TRLs play an important role in increasing the risk of atherogenesis (Bermúdez *et al*., 2008; Varela *et al*., 2013), while the dietary substitution of saturated fatty acids (SFA; mainly palmitic acid, 16:0) with monounsaturated fatty acids (MUFA; mainly oleic acid, 18:1 ω-9) influences protection against atherosclerosis by preventing excessive lipid accumulation in monocyte/macrophage cells (Varela *et al*., 2013). In our previous study, supplementation with pequi oil also reduced postprandial total cholesterol and low-density lipoprotein (LDL) in runners (particularly men) > 45 years old; LDL is considered an independent risk factor for CVD (Miranda-Vilela *et al*., 2009a). Since elevated plasma CRP and LDL have been associated with increased risk of CVD (Moleres *et al*., 2009), pequi oil has been suggested as a means of decreasing the risk of atherogenesis in these groups of more susceptible athletes (Miranda-Vilela *et al*., 2009a).

Despite these protective effects of pequi oil, some of the responses in runners are influenced by genetic polymorphisms related to oxidative stress and inflammatory markers (Miranda-Vilela *et al*., 2009b, 2010, 2011a,b; Ribeiro *et al*., 2013). Because some polymorphic genes are able to modify the risk of various diseases, and physical fitness has a genetic component, it would be interesting to study variations in genes that can influence athletic performance and pathogenic processes such as inflammatory responses (Colombini *et al*., 2011). In this regard, polymorphisms in the IL-6 gene need to be investigated better because they can influence performance, at least in those cases where the oxidative stress generated by strenuous exercise leads to muscle injuries and consequent inflammation.

The human IL-6 gene is located on the short arm of chromosome 7 (7p21) (Capurso *et al*., 2004) and has about 50 single-nucleotide polymorphisms (SNPs) in its promoter region (Pereira *et al*., 2011). Among these SNPs, a functional –174 G/C SNP (rs1800795) has been reported to affect the plasma levels of this cytokine, with the mutant C allele expressing lower levels of plasma IL-6 than the wild-type G allele (Fishman *et al*., 1998; Capurso *et al*., 2004). IL-6 regulates the immune response by acting on B and T cells, but also acts on hematopoietic stem cells, megakaryocytes and hepatocytes (e.g., mesangial cells), nerve cells, keratinocytes and plasmacytoma/myeloma cells (Hirano *et al*., 1990) such that changes in the expression of this cytokine can influence these cells. Indeed, a deficiency in IL-6 alters the balance between the proliferation and differentiation of progenitor cells of the granulocytic-monocytic, megakaryocytic and erythroid lineages into mature blood cells, leading to abnormal levels of committed progenitors in these lineages and to a slow recovery from hematopoietic ablation (Bernad *et al*., 1994).

Because the IL-6 –174 G/C polymorphism (SNP rs1800795) has been associated with exercise-related phenotypes (Eynon *et al*., 2011a,b), and since diet can affect an individual's genes and these can in turn affect the response to supplementation (Miranda-Vilela *et al*., 2011a; Miranda-Vilela, 2012; Ribeiro *et al*., 2013), in this work we investigated the influence of this IL-6 polymorphism on the levels of creatine kinase (CK), aspartate aminotransferase (AST), alanine aminotransferase (ALT), acute phase proteins (C-reactive protein - CRP and high-sensitivity CRP – hs-CRP), lipid peroxidation (evaluated by the TBARS assay), complete hemogram, and lipid profile of runners before and after ingestion of 400 mg of pequi oil in capsules supplied daily for 14 consecutive days. Overall, we sought to evaluate how individual genetic differences in IL-6 –174 G/C affected each athletes response to antioxidant supplementation with pequi oil during oxidative stress while exercising, and how the diet with pequi oil interacted with an individual's IL-6 gene to influence the response to this supplementation.

## Materials and Methods

### Study design and participants

Initially, 139 trained street runners of both genders (53 females and 86 males) and different age groups were recruited based on previously reported criteria (Miranda-Vilela *et al*., 2009a,b, 2010, 2011a,b; Ribeiro *et al*., 2013). Briefly, the tests were done after two races in the same environment and under closely comparable conditions, according to the type, intensity and length of the athletes weekly training, before and after the ingestion of 400 mg of pequi oil in capsules supplied daily for 14 consecutive days. There were no significant changes in the daily routine, training or lifestyle of the runners between the first and second race, except for the ingestion of pequi oil capsules. The athletes could choose the distance that they would cover (4-21 km) based on the type, intensity and length of their weekly training so as to guarantee no additional physical stress beyond that which they were accustomed to. This approach avoided differences in the amount or intensity of training and consequent increase in oxidative stress. Only those athletes who followed the instructions correctly and participated in both races were enrolled in the study, which involved 125 athletes (49 females and 76 males), aged 15-67 years old.

This study was done according to the guidelines laid down in the Declaration of Helsinki, and the procedures were approved by the Ethics Committee of the University of Brasília and by the National Commission for Ethics in Research (CONEP). Written informed consent was obtained from all subjects.

### Preparation of capsules

Pequi oil, the composition of which has previously been described (Miranda-Vilela *et al*., 2009a,b,c, 2010; Miranda-Vilela *et al*., 2011a,b; Ribeiro *et al*., 2013; Miranda-Vilela *et al*., 2014), was extracted by cold maceration using chloroform as a solvent (Miranda-Vilela *et al*., 2009c) and incorporated in Aerosil (colloidal silicon dioxide) q.s.p. (Miranda-Vilela *et al*., 2009a,b, 2010, 2011a,b). The capsule production was patented as number PI0601631-6 (National Institute of Industrial Property – INPI) and a voucher of the pequi specimen (*C. brasiliense* Camb.) was deposited in the herbarium of the University of Brasilia (UnB) by Professor Cassia Munhoz (PhD) (collection number 7402, registration number 165.857).

### Procedures and measurements

Waist circumference (WC), hip circumference, waist-hip ratio and body mass index (BMI) were checked before the first race as previously reported (Miranda-Vilela *et al*., 2009a). Peripheral blood samples collected immediately after the two races in Vacutainer tubes containing EDTA were used to perform immune cell counting and genotyping, while serum samples were used to quantify CK, AST, ALT, acute phase proteins (CRP and hs-CRP), postprandial lipid profile and TBARS.

### Hemogram and biochemical analyses

A complete blood count or hemogram was done in an automated analyzer (Cell-Dyn 3700, Abbott Diagnostics, Chicago, Illinois, US); serum ALT, AST, CK, CRP and postprandial lipid profile analyses were run on an automated chemistry analyzer ADVIA 1650 (Bayer Diagnostics, Greenburgh, NY, US) and serum hs-CRP was measured by an immunometric assay (Immulite 2000, DPC, Medlab) using the appropriate chemical reagents, controls and protocols of the manufacturers. The TBARS assay was done according to Wasowicz *et al*. (1993) and the fluorescence was measured with a Jasco FP-777 spectrofluorometer (excitation: 525 nm, emission: 547 nm).

### Genotyping of the polymorphism

Genomic DNA was isolated from the buffy-coat layer using a Blood genomic Prep mini spin kit (GE Healthcare, Buckinghamshire, England). DNA samples were quantified in a Nanovue spectrophotometer (GE Healthcare), diluted in milli-Q water to a final concentration of 50 ng/μL and stored at -20°C until analysis. DNA samples were amplified in an MJ PTC-100 thermocycler (MJ Research Inc., Waltham, MA, USA). The IL-6 genotypes were determined by allele-specific amplification (Eynon *et al*., 2011b). The PCR products were separated by electrophoresis in 6% non-denaturing polyacrylamide gels and visualized by staining with silver nitrate.

### Statistical analyses

The minimum sample size was estimated by power analysis based on the statistical analysis of quantitative data and a maximum tolerable sampling error (standard error or sampling error) of 0.05-0.20, depending on the population variability for the reference intervals of the laboratory tests and samples after stratification of the entire group (Ribeiro *et al*., 2013; Barbosa *et al*., 2014).

The genotype distributions were tested for Hardy-Weinberg equilibrium (HWE) by the Chi-square (χ^2^) test, using the Genepopweb statistical program, version 4.2 (http://genepop.curtin.edu.au). Values of p > 0.05 were indicative of HWE. The same program was used to calculate the allelic and genotypic frequencies of each locus, as well as genetic diversity parameters such as observed heterozygosity (Ho), expected heterozygosity (He) and inbreeding coefficient (FIS).

Statistical analysis was done using SPSS (Statistical Package for the Social Sciences) version 17.0. The data were expressed as the mean ± SD (standard deviation) and values of p < 0.05 were considered statistically significant. The continuous variables were tested for normal distribution with the Shapiro-Wilk test. For the parameters analyzed, possible differences between the sexes were evaluated by Students *t*-test or the Mann-Whitney U test (non-normalized data), while differences among age groups, distance covered and genotypes were evaluated by ANOVA or the Kruskal-Wallis test (data not normally distributed), followed, respectively, by the Tukey or Mann-Whitney U tests. Students paired *t*-test or the Wilcoxon matched pairs test (when the data were not normally distributed) was used to assess differences in before-after comparisons of supplementation with pequi oil.

The possible correlations between the parameters genetic polymorphisms/sex, genetic polymorphisms/age group and genetic polymorphisms/distance covered were analyzed using the Chi-square correlation test. As the correlations sex/age group, sex/distance covered, age group/distance covered have already been published (Miranda-Vilela *et al*., 2009a; Ribeiro *et al*., 2013), they will not be presented here. The Spearman correlation test was used to assess correlations between qualitative variables (genotypes) and laboratory tests, while correlations between quantitative variables were tested by the Pearson (normalized data) or Spearman (data not normally distributed) correlation tests (Barbosa *et al*., 2014).

The odds ratio (OR) with 95% confidence intervals (CI) was also calculated to estimate the relative chance of risk or protection for higher levels of CK, AST, ALT, CRP, hs-CRP and lipid peroxidation. To calculate the OR for the biochemical tests, the parameters > or < than the maximum reference limit were considered, and were: CK: 145 U/L (female) and 170 U/L (male) (Freire *et al*., 2008; Schumann and Klauke, 2003), AST: 31 U/L (female) and 37 U/L (male), ALT: 35 U/L (female) and 40 U/L (male) (Freire *et al*., 2008), and CRP and hs-CRP: 1.0 mg/L for both sexes, based on the low risk of having a heart attack as defined by the American Heart Association and the US Center for Diseases Control (Ridker, 2003), with women usually having lower values than men (Rifai and Ridker, 2003). For the TBARS assay the median was used, i.e., > 0.027 and < 0.027 nmol of MDA/mL for both sexes (Akimoto *et al*., 2010).

## Results

The frequencies of the IL-6 –174 G/C (SNP rs1800795) genotypes were in Hardy-Weinberg equilibrium (p > 0.05) and the distribution of their allele and genotype frequencies, as well as the genetic diversity parameters and HWE data for the Chi-square (χ2) test are shown in Table 1. There were no significant differences in the distribution of IL-6 genotypes between the sexes (Table 2), among age groups (Table 3) or in relation to the distance covered (Table 4).

**Table 1:** Distribution of IL-6 –174 G/C (SNP rs1800795) allele frequencies, genetic diversity parameters, genotype frequencies and Hardy-Weinberg equilibrium (HWE) data for the Chi-square (χ^2^) test.

Genetic polymorphism	Chromosome location	Allele frequencies		Heterozygosity-observed (H_o_)	Heterozygosity-expected (H_e_)	F_IS_ (Inbreeding coefficient)	Genotypes	Genotype frequencies	Number of observed individuals	Number of expected individuals	HWE test (p)
IL-6 -174	7p15.3	G	0.68				GG	0.4720	59	57.6908	
G/C		C	0.32	0.4160	0.4352	0.0481	GC	0.4160	52	54.6185	0.6806
							CC	0.1120	14	12.6908	

The p value was calculated using the statistical program Genepopweb version 4.2 (http://genepop.curtin.edu.au).

**Table 2:** Distribution of IL-6 –174 G/C (SNP rs1800795) genotypes in relation to the total number of subjects and gender. The results are expressed as a percentage (%) in relation to the total sample size of each group.

IL-6 genotypes	Total (%) [N=125]	Male (%) [N=76]	Female (%) [N=49]	p
GG	59 (47.2)	38 (50)	21 (42.9)	
GC	52 (41.6)	29 (38.2)	23 (46.9)	0.569
CC	14 (11.2)	9 (11.8)	5 (10.2)	

The p value was calculated with the Mann-Whitney U test using SPSS (*Statistical Package for the Social Sciences*), version 17.0.

**Table 3:** Distribution of IL-6 –174 G/C (SNP rs1800795) genotypes in relation to age group (years old). The results are expressed as a percentage (%) in relation to the total sample size of each group.

IL-6 genotypes	15-19 (%) [N=20]	20-24 (%) [N=25]	25-29 (%) [N=25]	30-34 (%) [N=12]	35-39 (%) [N=16]	40-44 (%) [N=10]	> 45 (%) [N=17]	p
GG	10 (50)	10 (40)	14 (56)	4 (33.3)	9 (56.3)	4 (40)	8 (47.1)	
GC	5 (25)	10 (40)	10 (40)	7 (58.3)	6 (37.5)	6 (60)	8 (47.1)	0.717
CC	5 (25)	5 (20)	1 (4)	1 (8.3)	1 (6.3)	0 (0)	1 (5.9)	

The p value was calculated with the Kruskal-Wallis test using SPSS (*Statistical Package for the Social Sciences*), version 17.0.

**Table 4:** Distribution of IL-6 –174 G/C (SNP rs1800795) genotypes relative to the distance covered (km). The results are expressed as a percentage (%) in relation to the total sample size of each group.

IL-6 genotypes	4-5 (%) [N=50]	6-7 (%) [N=38]	8-10 (%) [N=30]	16-21 (%) [N=7]	p
GG	23 (46)	21 (55.3)	11 (36.7)	4 (57.1)	
GC	19 (38)	13 (34.2)	18 (60)	2 (28.6)	0.697
CC	8 (16)	4 (10.5)	1 (3.3)	1 (14.3)	

The p value was calculated with the Kruskal-Wallis test using SPSS (*Statistical Package for the Social Sciences*), version 17.0.

For the biochemical tests, there were significant differences in the TBARS values of the genotypes CC and GG (p=0.011) and CC and GC (p=0.028) before supplementation with pequi oil. After supplementation, these differences persisted between CC and GG (p=0.023) and appeared for GC and GG (p=0.041). In both cases, before and after pequi, the wild type (GG) genotype showed higher lipid peroxidation [higher MDA (malondialdehyde) values in the TBARS assay]. Significant differences in the before-after comparison were observed for the GC genotype in relation to the CK (p = 0.030) and AST (p = 0.030) values that were reduced after supplementation, and for the CC genotype in which CRP was significantly increased (p = 0.021) after supplementation with pequi, although still within the limits of the reference value (Ridker, 2003) (Table 5).

**Table 5:** Influence of IL-6 –174 G/C polymorphism (SNP rs1800795) on the CK, AST, ALT, CRP, hs-CRP and TBARS values before and after supplementation with pequi oil.

	CK (U/L)		AST (U/L)		ALT (U/L)		CRP (mg/dL)		hs-CRP (mg/dL)		TBARS (nmol of MDA/mL)	
IL-6 genotypes	Before	After	Before	After	Before	After	Before	After	Before	After	Before	After
GG	327.84 ± 425.25	275.39 ± 295.09	29.98 ± 8.47	29.26 ± 7.55	23.60 ± 11.23	21.91 ± 8.96	0.37 ± 0.41	0.37 ± 0.32	1.95 ± 2.81	1.78 ± 1.93	0.0280 ± 0.008	0.0278 ± 0.006
GC	237.75 ± 217.36	186.08 ± 106.10^#^	30.04 ± 8.88	27.52 ± 6.49^#^	23.19 ± 11.52	21.94 ± 9.48	0.31 ± 0.23	0.33 ± 0.25	1.32 ± 1.54	1.29 ± 1.64	0.0264 ± 0.007	0.0257 ± 0.006^a^
CC	271.71 ± 307.44	260.64 ± 212.95	28.36 ± 9.88	26.43 ± 8.47	20.57 ± 11.92	19.14 ± 7.50	0.21 ± 0.18	0.52 ± 0.39^#^	1.06 ± 0.99	1.66 ± 1.91	0.0222 0.007^a,b^	0.0229 ± 0.007^a^
p	0.192	0.428	0.494	0.274	0.271	0.519	0.371	0.247	0.291	0.194	0.026	0.019

The results are expressed as the mean ± SD. ALT – alanine aminotransferase, AST – aspartate aminotransferase; CK – creatine kinase, CRP – C-reactive protein, hs-CRP – high-sensitivity CRP, U/L – units per liter, mg/dL – milligrams per deciliter, nmol of MDA/mL – nanomoles of malondialdehyde per milliliter of serum. The p values were calculated using the Kruskal-Wallis test. The lowercase letters indicate significant differences detected by the Mann-Whitney U test between genotypes in 2 X 2 comparisons, where a and b indicate significance compared to the GG and GC genotypes, respectively. The symbol # indicates significant differences in the comparisons before and after supplementation with pequi oil as detected by the Wilcoxon test.

For the hemogram, supplementation with pequi resulted in a significant difference only for the platelet deviation weight (PDW) between the genotypes GC and GG (p = 0.045), with the heterozygous genotype having the higher values. However, in the before-after comparison, several responses to supplementation were observed among the IL-6 genotypes (Table 6).

**Table 6:** Influence of IL-6 –174 G/C polymorphism (SNP rs1800795) on erythrocytes (A), leukocytes (B) and platelets (C) before and after supplementation with pequi oil.

Erythrocytes														
IL-6 genotypes	RBC (millions/mm^3^)		HGB (g/dL)		HCT (%)		MCV (fl)		MCH (pg)		MCHC (g/% ou g/dL)		RDW (%)	
	Before	After	Before	After	Before	After	Before	After	Before	After	Before	After	Before	After
GG	5.20 ± 0.50	5.14 ± 0.50	14.82 ± 2.24	14.76 ± 2.25	44.47 ± 3.60	43.86 ± 3.65^#^	85.74 ± 4.19	85.73 ± 4.28	29.25 ± 1.59	29.57 ± 1.62^#^	34.13 ± 0.98	34.50 ± 0.58^#^	14.92 ± 1.09	14.28 ± 1.18^#^
GC	5.25 ± 0.53	5.17 ± 0.47^#^	14.40 ± 3.21	14.37 ± 3.26	45.41 ± 4.03	44.73 ± 3.62	86.58 ± 3.29	86.69 ± 3.09	29.53 ± 1.55	29.96 ± 1.40^#^	34.10 ± 1.08	34.55 ± 0.69^#^	14.86 ± 0.84	14.33 ± 0.96^#^
CC	5.26 ± 0.46	5.16 ± 0.54	13.37 ± 4.45	13.11 ± 4.45^#^	45.77 ± 3.33	44.84 ± 3.84^#^	87.12 ± 3.49	87.14 ± 4.13	30.06 ± 1.23^a^	30.09 ± 1.43	34.49 ± 0.72	34.57 ± 0.83	14.47 ± 1.11	13.89 ± 0.93^#^
p	0.859	0.953	0.917	0.717	0.320	0.415	0.310	0.315	0.098	0.251	0.555	0.557	0.321	0.386

Leukocytes														
IL-6 genotypes	WBC (/mm^3^)		Lymphocytes (/mm^3^)		Segmented (/mm^3^)		Rods (/mm^3^)		Basophils (/mm^3^)		Eosinophils (/mm^3^)		Monocytes (/mm^3^)	
	Before	After	Before	After	Before	After	Before	After	Before	After	Before	After	Before	After
GG	7479.63 ± 2265.44	7455.56 ± 1918.79	2595.07 ± 993.02	2519.36 ± 927.72	4099.18 ± 1712.91	4097.45 ± 1431.03	45.33 ± 114.77	36.22 ± 101.18	83.82 ± 49.08	95.64 ± 50.05	150.76 ± 115.62	159.96 ± 131.14	509.67 ± 248.69	550.20 ± 211.13
GC	7428.57 ± 2504.83	7508.16 ± 1916.90	2757.73 ± 967.59	2762.43 ± 965.14	3942.14 ± 1918.00	3851.43 ± 1427.28	9.81 ± 29.72	14.23 ± 56.91	86.67 ± 52.35	99.10 ± 43.88	148.24 ± 125.84	164.92 ± 132.83^#^	481.14 ± 198.17	557.20 ± 201.21^#^
CC	7371.43 ± 1219.35	7264.29 ± 2259.55	2666.36 ± 919.23	2552.79 ± 1367.59	3963.64 ± 1145.49	3938.43 ± 1629.83	7.57 ± 28.33	31.00 ± 90.22	87.64 ± 56.75	107.57 ± 46.38	122.79 ± 82.68	108.57 ± 63.06	503.29 ± 197.44	517.07 ± 216.85
p	0.842	0.843	0.603	0.451	0.536	0.711	0.223	0.557	0.947	0.695	0.763	0.384	0.850	0.728

Platelets								
IL-6 genotypes	Platelets (thousand/mm^3^)		Platelets (%)		MPV (fl)		PDW (%)	
	Before	After	Before	After	Before	After	Before	After
GG	337.54 ± 72.86	309.07 ± 61.22^#^	0.36 ± 0.10	0.31 ± 0.07^#^	10.42 ± 1.59^#^	10.03 ± 1.45	18.07 ± 1.16	17.86 ± 0.92
GC	336.92 ± 67.49	319.76 ± 69.30^#^	0.36 ± 0.09	0.35 ± 0.10	10.59 ± 1.68	10.67 ± 1.79	17.87 ± 1.08	18.35 ± 1.11^#^
CC	320.43 ± 61.78	297.93 ± 61.07	0.34 ± 0.06	0.32 ± 0.07	10.83 ± 1.62	10.75 ± 1.82	18.23 ± 1.47	18.29 ± 1.15
p	0.544	0.505	0.722	0.376	0.593	0.199	0.641	0.123

The data are expressed as the mean ± SD. HCT – hematocrit, HGB – hemoglobin, MCV – mean corpuscular volume, MCH – mean corpuscular hemoglobin, MCHC – mean corpuscular hemoglobin concentration, MPV – mean platelet volume, PDW – platelet deviation weight, RBC – red blood cells, RDW – red cell distribution width, WBC – white blood cells, g/dL – gram per deciliter, fl – femtoliter, pg – picograms, g/% – gram per percentage. P values for RBC, HCT, RDW and basophils were calculated by ANOVA, while the other p values were calculated with the Kruskal-Wallis test. The lowercase letters indicate significant differences between genotypes in the 2 X 2 comparisons, where a and b indicate significance compared to the GG and GC genotypes, respectively. The symbol # indicates significant differences in the comparisons before and after supplementation with pequi oil detected by Students *t*-test (HCT, RBC, RDW) or the Wilcoxon test (other variables).

The postprandial lipid profile revealed significant differences after supplementation with pequi only between the LDL values of the CC and GG genotypes (p=0.012) and CC and GC (p=0.003), with the homozygous mutant genotype (CC) having lower values. No significant differences were observed in the before-after comparisons (Table 7).

**Table 7:** Influence of IL-6 –174 G/C polymorphism (SNP rs1800795) on the postprandial lipid profile before and after supplementation with pequi oil.

IL-6 genotypes	Total cholesterol (mg/dL)		Triglycerides (mg/dL)		HDL (mg/dL)		LDL (mg/dL)		VLDL (mg/dL)	
	Before	After	Before	After	Before	After	Before	After	Before	After
GG	187.37 ± 37.43	186.04 ± 35.98	116.06 ± 66.43	116.72 ± 55.42	54.02 ± 13.90	53.07 ± 13.80	109.68 ± 28.56	108.85 ± 27.77	23.21 ± 13.29	23.34 ± 11.08
GC	193.22 ± 39.60	193.29 ± 33.71	111.35 ± 55.53	116.41 ± 46.27	55.14 ± 12.87	55.98 ± 13.32	116.19 ± 35.36	113.91 ± 29.26	22.27 ± 11.11	23.28 ± 9.25
CC	169.57 ± 35.67	170.21 ± 32.63	108.21 ± 58.52	130.86 ± 63.45	51.93 ± 12.99	54.50 ± 16.22	96.00 ± 26.19	89.54 ± 21.75a,b	21.64 ± 11.70	26.17 ± 12.69
p	0.126	0.086	0.945	0.747	0.368	0.313	0.149	0.012	0.945	0.747

The data are expressed as the mean ± SD. HDL – high-density lipoprotein, LDL – low-density lipoprotein, VLDL – very-low-density lipoprotein, mg/dL= milligram per deciliter. P values for total cholesterol were calculated by ANOVA, while those for the other parameters were calculated by the Kruskal-Wallis test. The lowercase letters indicate significant differences detected between genotypes in the 2 X 2 comparisons, where a and b indicate significance compared to the GG and GC genotypes, respectively.

Several correlations among the serum levels of the biochemical parameters and postprandial lipid profile were observed in the group as a whole and in the IL-6 genotypes. In particular, there was a positive correlation between triglycerides before vs hs-CRP, which was particularly related to the CC genotype (Table 8).

**Table 8:** Correlation between total cholesterol and other serum lipids in the whole group and in relation to the IL-6 –174 G/C genotypes.

Group	Comparison	Correlation	p
		coefficient	
Whole group	Total cholesterol before vs		
	Triglycerides before	0.366	0.000
	HDL before	0.409	0.000
	LDL before	0.905	0.000
	VLDL before	0.366	0.000
	Total cholesterol after vs		
	Triglycerides after	0.365	0.000
	HDL after	0.406	0.000
	LDL after	0.897	0.000
	VLDL after	0.365	0.000
	Triglycerides before vs		
	VLDL before	1.000	0.000
	hs-CRP before	0.197	0.041
	Triglycerides after vs		
	HDL after	-0.182	0.049
	VLDL after	1.000	0.000
IL-6 GG genotype	Total cholesterol before vs		
	Triglycerides before	0.444	0.003
	LDL before	0.554	0.000
	VLDL before	0.463	0.002
	Total cholesterol after vs		
	Triglycerides after	0.432	0.004
	HDL after	-0.304	0.045
	LDL after	0.451	0.002
	VLDL after	0.431	0.004
	Triglycerides before vs		
	HDL before	-0.363	0.017
	VLDL before	1.000	0.000
	Triglycerides after vs		
	HDL after	-0.426	0.004
	VLDL after	1.000	0.000
	HDL before vs		
	VLDL before	-0.363	0.017
	HDL after vs		
	VLDL after	-0.426	0.004
IL-6 GC genotype	Total cholesterol before vs		
	Triglycerides before	0.318	0.031
	LDL before	0.544	0.000
	VLDL before	0.318	0.031
	Total cholesterol after vs		
	LDL after	0.548	0.000
	Triglycerides before vs		
	VLDL before	1.000	0.000
	Triglycerides after vs		
	VLDL after	1.000	0.000
IL-6 CC genotype	Triglycerides before vs		
	VLDL before	1.000	0.000
	hs-CRP before	0.843	0.001
	Total cholesterol after vs		
	LDL after	0.756	0.018
	Triglycerides after vs		
	VLDL after	1.000	0.000

The OR with 95% CI indicated that individuals carrying the wild type genotype (GG) were 2.9 times more likely to have MDA values (TBARS assay) > 0.027 nmol/mL, while the GC genotype decreased this risk. For females carrying the GG genotype, this risk was > 5.0, while for males, there was a decreased risk for the CC, but not for GC, genotype (Table 9).

**Table 9:** Odds ratios (OR) and 95% confidence intervals (CI).

		Comparisons	OR (95% CI)	p
Total group		IL-6 GG and TBARS after > 0.027 nmol of MDA/mL	2.917 (1.407-6.047)	0.004
		IL-6 GC and TBARS after > 0.027 nmol of MDA/mL	0.462 (0.222-0.961)	0.046
Gender	Male	IL-6 CC and TBARS before > 0.027 nmol of MDA/mL	0.129 (0.015-1.087)	0.031
	Female	IL-6 GG and TBARS after > 0.027 nmol of MDA/mL	5.278 (1.535-18.148)	0.007
		IL-6 GC and TBARS after > 0.027 nmol of MDA/mL	0.232 (0.070-0.770)	0.015

## Discussion

The IL-6 –174 G/C polymorphism (SNP rs1800795) tends to be quite variable in Caucasians, but in Asians and Africans the frequency of the C allele is much lower than in Caucasians, tending to be almost monomorphic for the wild-type G allele (Gan *et al*., 2013). The Brazilian population is very mixed, primarily because of five centuries of interethnic crosses among Europeans (European colonizers, mainly represented by the Portuguese), Africans (slaves) and Amerindians (the indigenous population) (Parra *et al*., 2003; Hiragi *et al*., 2011; Lordelo *et al*., 2012; Barbosa *et al*., 2014); this miscegenation can strongly influence the distribution of certain polymorphisms (Lordelo *et al*., 2012; Barbosa *et al*., 2014). Moreover, Brazil's large geographic size and the fact that different population groups have moved to different parts of the country has resulted in considerable phylogeographical heterogeneity (Parra *et al*., 2003). As the population of Brasilia (the Federal Capital) is formed by migrants from all regions of Brazil, it tends to reflect the Brazilian population better than any other region (Miranda-Vilela *et al*., 2009d; Hiragi *et al*., 2011). In this regard, our results confirm a major influence of European ancestry in the IL-6 polymorphism studied here. Moreover, as there were no sex-, age- or distance-related differences in the distribution of the IL-6 genotypes, we have no reason to reject the hypothesis that the significant differences seen here reflected each individuals IL-6 response to antioxidant supplementation with pequi, and possibly also a direct interaction of pequi with the IL-6 gene to influence the response to this supplementation.

Although we did not examine the biomarkers before each race, most of the exercise-induced physiological and biochemical changes have already been well documented (Ji and Leichtweis, 1997; Kargotich *et al*., 1998; Kasapis and Thompson, 2005; Mattusch *et al*., 2000; Urso and Clarkson, 2003; Brancaccio *et al*., 2007; Cruzat *et al*., 2007; Ferreira *et al*., 2007; Mougios, 2007) and our study did not aim to evaluate such alterations. We undertook a controlled before-after study in which observations are made before and after the implementation of an intervention; in our case, before (first race) and after (second race) intervention with pequi oil. This type of study is validated in the scientific literature (Meads and Davenport, 2009) and, although it has some limitations compared to randomized placebo-controlled studies, we followed all of the recommended steps to guarantee quality control and the validity of our study (American College of Physicians, 1999; Meads and Davenport, 2009), as previously reported (Miranda-Vilela *et al*., 2009a,b, 2010, 2011a,b; Ribeiro *et al*., 2013). Our results support the proposed hypothesis since the values observed did not exceed the reference limits determined for clinical purposes (Ridker, 2003; Schumann and Klauke, 2003; Freire *et al*., 2008), and much less for athletes (Mougios, 2007).

In addition to increasing oxygen consumption and inducing oxidative stress, exercise can initiate an inflammatory process that is regulated by cytokines, mostly IL-6 (Moleres *et al*., 2009). Since the IL-6 –174 G/C polymorphism is associated with serum IL-6 and/or CRP levels (Szydlowski *et al*., 2013), our results demonstrated that pequi oil may remove the positive association between triglycerides and hs-CRP seen in the homozygous mutant IL-6 CC genotype before (but not after) pequi. Furthermore, pequi oil may promote a non-significant increase in triglycerides (Miranda-Vilela *et al*., 2009a) because of its natural triglyceride (TG) composition (Segall *et al*., 2006; Miranda-Vilela *et al*., 2009c). Since individuals genetically predisposed to higher IL-6 secretion may be at risk of dyslipidemia, especially during the postprandial phase, it has been suggested that the IL-6 –174 G/C polymorphism determines the difference in both fasting and postprandial TG metabolism and that this phenomenon could be responsible for the observed association of this genetic variant with a risk for CVD (Shen *et al*., 2008). C-reactive protein (CRP) is an acute phase reactant and indicator of inflammation that promotes lipid accumulation in the atherosclerotic plaque and exerts direct effects on endothelial cells, thereby contributing to endothelial dysfunction (Erbel *et al*., 2008). CRP and hs-CRP measure the same molecule in blood, but hs-CRP has been developed to detect CRP at lower levels and is therefore much more sensitive for diagnostic purposes (Rifai and Ridker, 2003). The levels of hs-CRP can be used to predict future cardiovascular disease in seemingly healthy middle-aged adults (Erbel *et al*., 2008), and the lack of correlation after pequi supplementation in the present study suggested a protective effect of pequi oil, primarily for the IL-6 CC genotype.

Pequi oil has a high concentration of monounsaturated oleic (MUFA) and saturated palmitic (SFA) fatty acids that are anti- and pro-atherogenic agents, respectively (Aguilar *et al*., 2012). This oil is also rich in natural antioxidants such as carotenoids (Azevedo-Meleiro and Rodriguez-Amaya, 2004; Oliveira *et al*., 2006; Lima *et al*., 2007) and vitamin E (α-tocopherol), both of which are encountered in cooked pulp (Cardoso *et al*., 2013). Thus, although pequi oil has been associated with atherogenic worsening of the lipid profile (Aguilar *et al*., 2012), it has also been reported to efficiently reduce exercise-induced inflammation (Miranda-Vilela *et al*., 2009a). This anti-inflammatory action may be associated not only with the antioxidant properties of the oil, but also with its MUFA oleic acid-to-SFA palmitic acid ratio.

The increase in O_2_ consumption induced by physical exercise is associated with an increase in reactive oxygen species (ROS) production. These species induce oxidative stress, a term generally used to describe the damage caused by an imbalance between pro-oxidants and antioxidant defense mechanisms (Leal Junior *et al*., 2011). Endothelial oxidative stress is associated with impaired function and is a key feature in the onset and evolution of CVD (Conti *et al*., 2012). The increase in the MUFA oleic acid-to-SFA palmitic acid ratio in postprandial TRL has been linearly correlated with an increased down-regulation of the apolipoprotein B48 receptor (ApoB48R; a macrophage receptor that binds to apolipoprotein B48 of dietary TRL), with a consequent decrease in lipid accumulation (Varela *et al*., 2013). This receptor may provide essential lipids, lipid-soluble vitamins and other nutrients to reticuloendothelial cells. When overwhelmed with elevated plasma triglyceride levels, the apolipoprotein B48 receptor may contribute to foam cell formation, endothelial dysfunction and atherothrombogenesis (Brown *et al*., 2000). These responses agree with the suggestion regarding the importance of the MUFA oleic acid-to-SFA palmitic acid ratio indicated above.

High concentrations of ROS result in damage to DNA, proteins and lipids that can cause cell and tissue impairment (Conti *et al*., 2012). Biomarkers of lipid oxidation, such as malondialdehyde (MDA) measured as thiobarbituric acid reactive substances (TBARS), may be independent risk indicators for patients with stable coronary artery disease (CAD), independently of traditional risk factors and inflammatory markers (Walter *et al*., 2004). In the present study done in athletes, the wild type GG genotype showed significantly higher MDA values than the other IL-6 genotypes, a situation that was not improved by supplementation with pequi oil. In addition, the Odds ratio indicated an increased risk for higher MDA values among females. Women have a lower risk of CVD than men because endogenous estrogens during the fertile period of life delay the manifestation of atherosclerotic disease in women (Maas and Appelman, 2010). In the present study, only athletes in their fertile period were investigated, with the overall mean absolute level of TBARS being much lower than that reported for patients with stable coronary artery disease (1.49 ± 0.57 μM or ng/dL) (Walter *et al*., 2004). In addition, the absolute global CVD risk inspired by the Framingham Heart Study is calculated based on a combination of several key risk factors that include the patients history of cardiovascular disease, diabetes, smoking, blood pressure and blood lipid concentrations (Bitton and Gaziano, 2010). Pequi oil reportedly modulates postprandial lipemia and reduces exercise-induced inflammation and blood pressure in runners (Miranda-Vilela *et al*., 2009a).

Exercise induces transitory alterations in the serum/plasma cytokine profile that involve mainly an increase in serum levels of interleukin-6 (IL-6) (Oleto *et al*., 2011). Although we did not quantify circulating IL-6 levels, the significant reductions in CK and AST seen in individuals heterozygous for IL-6 and the non-significant reduction in these parameters observed for the other genotypes in the before-after comparison of pequi-oil suggested a better response to this antioxidant supplementation against exercise-induced damage in the GC genotype. The results of the platelet count corroborate our suggestion.

In conclusion, the IL-6 genotypes showed significant differences in lipid peroxidation, with the CC mutant showing lower values. There were also significant differences among the genotypes with respect to the response to antioxidant supplementation with pequi oil, mainly in relation to exercise-induced damage and CRP levels. The best response against muscle damage was seen in the heterozygous genotype, although the CC genotype showed an increase in CRP levels after supplementation; the lack of a positive correlation between triglycerides and hs-CRP for this mutant genotype after supplementation indicated a protective effect of pequi oil. Because pequi oil has been associated with an atherogenic effect, worsening the lipid profile and at the same time modulating posprandial lipemia and reducing exercise-induced inflammation and blood pressure of human runners, these findings deserve further investigations, in which evaluations of the IL-6 levels should also be performed.
